# Supplementary material for: Expression and Prognostic Evaluation of the Receptor Tyrosine Kinase MET in Canine Malignant Melanoma
Source: Vet Sci. 2023 Mar 26;10(4):249. doi: 10.3390/vetsci10040249 (PMC10144085; doi:10.3390/vetsci10040249)
Supplement: Supplementary file 1 [file vetsci-10-00249-s001.zip › vetsci-2293192-supplementary.pdf]

Supplemental Materials

Table S1. Summary of Relevant Clinical Data for Included 30 Cases

Study ID	Gender[1]	Breed	Weight (kg)[2]	Age (year)[2]	Tumor type	Anatomic location	Met Score	LN metastasis[3]	Distant metastasis	Treatment	Surival time (days)
C01	FS	Rottweiler	34.0	2	Accral	L front 5th digit	1	Y	Y	Sx	Lost to follow-up
C02	FS	German Shepherd	29.4	13	Oral	Palatal to left maxillary canine (205)	0	N	N	Sx and Vaccine	70
C03	FS	Labrador Retriever	28.4	14	Oral	R mandibular lingual mucosa between PM1 and M1	1	N	N	Sx and RT	288
C04	FS	Shar Pei	28.1	15	Oral	Lingual - R dorsal surface	0	Y - Suspected	Y	RT	322
C05	FS	Shih Tzu	5.6	9	Cutaneous	dermal/ SQ L perianal mass	0	N	N	Sx and RT	329
C07	FS	Giant Schnauzer	42.5	11	Cutaneous	R lateral antebrachium	1	N	N	None	Lost to follow-up
C08	MN	Mixed	37.6	8	Oral	R lateral mandible around 408	2	N	N	Sx and Vaccine	Lost to follow-up
C09	FS	Labrador Retriever	23.1	15	Accral	L hind 3rd digit	1	N	Y	Sx and Vaccine	Lost to follow-up
C10	FS	Miniature Schnauzer	9.6	8	Accral	R front 3rd digit	2	N	Y	Sx and Vaccine	829

Study ID	Gender[1]	Breed	Weight (kg)[2]	Age (year)[2]	Tumor type	Anatomic location	Met Score	LN metastasis[3]	Distant metastasis	Treatment	Surival time (days)
C11	FS	Standard Poodle	24.4	11	Accral	L hind 2nd digit	0	N	N	Sx and Vaccine	Lost to follow-up
C15	MN	Golden Retriever	33.8	12	Oral	Gingiva around 208 and 209	1	N	Y - Suspected[4]	Sx and RT	463
C16	MI	Labrador Retriever	25.4	12	Oral	Caudal to 109/110	0	N	N	Sx and Vaccine	657
C17	FS	German Shepherd	28.0	10	Cutaneous	R metacarpal pad	1	N	Y	Sx and Vaccine	Lost to follow-up
C18	MN	Scottish Terrier	12.3	11	Oral	R upper lip mucosa	1	N	N	Sx	1023
C21	MN	Basset Hound	36.7	12	Oral	R maxillary gingiva above 106	1	N	N	None	41
C22	MN	Golden Retriever	40.8	7	Oral	Sublingual	0	Y - Suspected	N	Sx and Vaccine	Lost to follow-up
C23	FS	Gordon Setter	14.7	9	Oral	Mucocutaneous junction of R maxillary lip	1	N	N	Sx and Vaccine	Lost to follow-up
C24	MN	Bernese Mountain Dog	47.2	7	Cutaneous	Ventral L thorax at the level of the 8th intercostal space	0	N	N	Sx, Vaccine, and Chemo	Lost to follow-up
C25	FS	Gordon Setter	29.0	11	Oral	Gingiva on lingual aspect of 408 and 409	1	Y - Suspected	Y	Sx, Vaccine, and Chemo	193

Study ID	Gender[1]	Breed	Weight (kg)[2]	Age (year)[2]	Tumor type	Anatomic location	Met Score	LN metastasis[3]	Distant metastasis	Treatment	Surival time (days)
C26	FS	Saint Bernard	68.8	11	Oral	L mandibular lip	0	N	Y	Sx, Vaccine, and Chemo	58
C27	FS	Labrador Retriever	26.2	12	Oral	L mandibular buccal gingiva	0	Y	N	Sx and RT	434
C28	MN	Labrador Retriever	39.6	13	Oral	Rostral mand lingual region around incisors	0	Unknown	Unknown	None	Lost to follow-up
C29	FS	Yorkshire Terrier	3.2	13	Oral	L mandible extending from 305 caudally	0	N	Y - Suspected[5]	Sx and RT	130
C30	MN	Golden Retriever	37.1	10	Oral	dorsolateral pharynx near tonsil, along R mandibular ramus involving palate	1	N	Y	RT	455
C32	MN	Pit Bull Terrier	40.4	8	Oral	R caudal maxilla	1	N	Y	Sx and RT	427
C33	FS	German Shepherd	41.6	13	Oral	Caudal soft palate	1	N	Y - Suspected[6]	Sx and RT	224
C34	MN	Labrador Retriever	39.7	10	Oral	R cd hard palate and soft palate in the region of 108 - 110	1	N	N	Sx and RT	Lost to follow-up
C35	MN	Giant Schnauzer	Unknown	4	Accral	R front digit 3	1	Unknown	Y	Sx and Vaccine	290

Study ID	Gender[1]	Breed	Weight (kg)[2]	Age (year)[2]	Tumor type	Anatomic location	Met Score	LN metastasis[3]	Distant metastasis	Treatment	Surival time (days)
C37	FS	German Shepherd	39.9	8	Cutaneous	L pinna - marginal cutaneous flap	1	Unknown	Unknown	Sx	Alive
C38	FS	Cocker Spaniel	7.1	9	Oral	L mandibular ginigva near 305 and 306	2	Unknown	Unknown	Sx and Vaccine	Alive

1. FS = female spayed; MN = male neutered; MI = male intact
2. Weight and age at time of diagnosis
3. Suspected metastasis from LN palpation without cytologic support
4. Lytic bone lesion on mid-diaphysis of L radius on radiographs
5. Cutaneous melanoma on punch biopy
6. Two round soft tissue opacities on thoracic radiographs may represent pulmonary metastasis or superimposition of structure

Table S2. Treatments elected among the 30 cases reviewed

Treatment	No. of Cases	% of Total Cases
**Surgery and vaccine**	11	36.70%
**Surgery and radiation therapy**	8	26.70%
**Surgery only**	3	10.00%
**Surgery, vaccine, and chemotherapy**	3	10.00%
**No treatment**	3	10.00%
**Radiation therapy only**	2	6.70%

Table S3. Univariate analysis of histologic features and Met scores for all canine melanomas

		Overall Met scores			
**Histologic Feature**	**Values**	**0**	**1**	**2**	**p-value**
**Degree of pigmentation (count)**	<50%	6	11	2	
	>50%	5	5	1	0.52
**Presence of ulceration (count)**	N	4	3	1	
	Y	7	13	2	0.57
**Presence of necrosis (count)**	N	8	13	2	
	Y	3	3	1	0.92
**Presence of deep inflammation (count)[1]**	N	6	6	1	
	Y	4	6	2	0.41
**Median mitotic index[2]**	--	3	3.5	2	0.95
**Median minimum Breslow thickness (mm)**	--	10	4.4	3.7	0.79

1. 5 cases (4 orals and 1 acral) were not able to be classified due to lack of inflammation or tumor extension to tissue margin
2. Mitotic index = mitotic count per 10 high power fields

Table S4. Univariate analysis of histologic features and Met scores for oral canine melanomas

		Oral melanoma Met scores			
**Histologic Feature**	**Values**	**0**	**1**	**2**	**p-value**
**Degree of pigmentation (count)**	<50%	4	7	1	
	>50%	4	3	1	0.67
**Presence of ulceration (count)**	N	3	0	1	
	Y	5	10	1	0.48
**Presence of necrosis (count)**	N	7	7	2	
	Y	1	3	0	0.86
**Presence of deep inflammation (count)[1]**	N	5	2	1	
	Y	2	5	1	0.27
**Median mitotic index[2]**	--	2	4.5	1	0.6
**Median minimum Breslow thickness (mm)**	--	9.5	4.7	3.4	0.86

1. 5 cases (4 orals and 1 acral) were not able to be classified due to lack of inflammation or tumor extension to tissue margin
2. Mitotic index = mitotic count per 10 high power fields

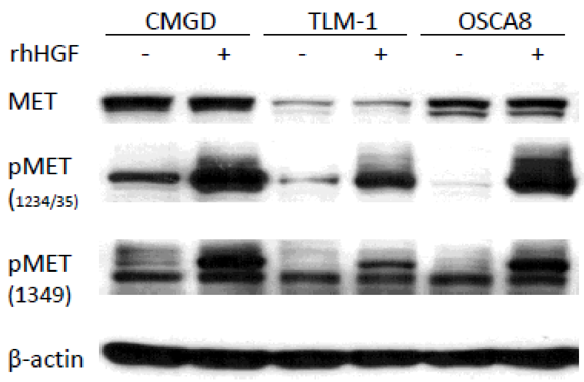

Figure S1. Recombinant human HGF (rhHGF) induces MET activation. CMGD-2 and TLM-1 are canine melanoma cell lines and OSCA-8 is a canine osteosarcoma cell line, used as positive control. All the experiments were performed after serum starvation and then stimulated with rhHGF (+) or untreated (-).

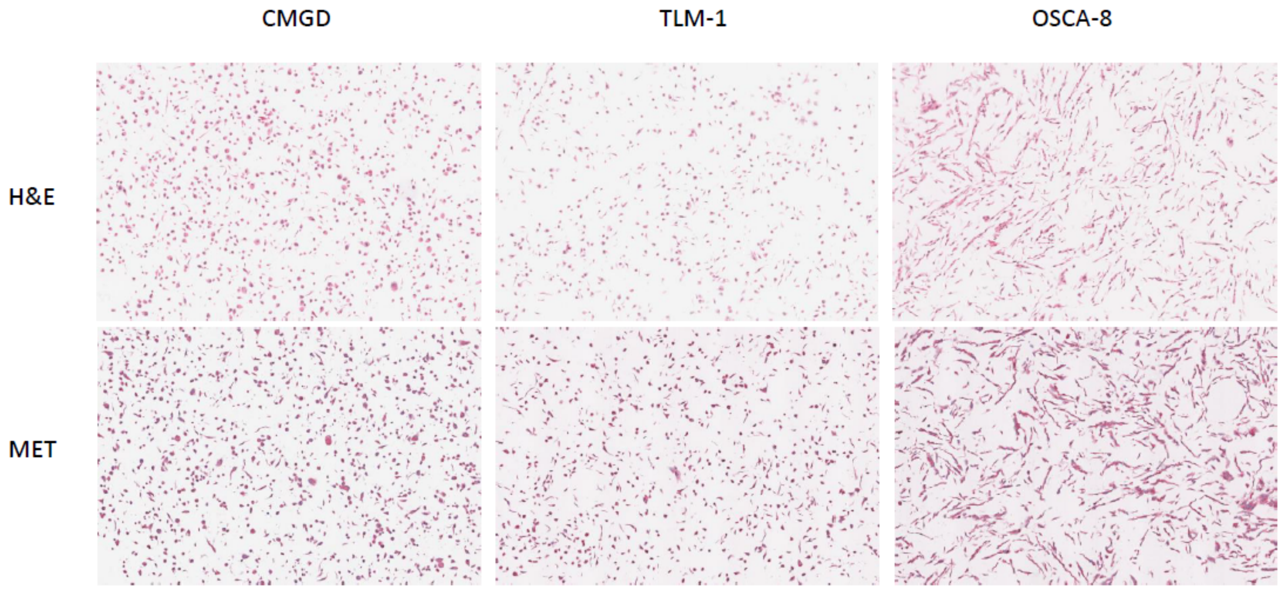

Figure S2. Confirmed immunopositivity with H&E staining and MET immunohistochemistry performed on cell pallets created from canine melanoma cell lines CMGD-2 and TLM-1 and canine osteosarcoma cell line OSCA-8 as positive control.
